# A One‐Year Visual and Safety Evaluation of a Newly Available Trifocal Intraocular Lens in Patients With Bilateral Cataracts: Clinical Outcomes From a Prospective Study

**DOI:** 10.1155/joph/5786325

**Published:** 2026-10-05

**Authors:** Peter Mojžiš, Andrea Janeková, Lucia Hrčková, Iveta Němcová, Philipp Eberwein

**Affiliations:** ^1^ Department of Ophthalmology Havlickuv Brod Hospital Havlickuv Brod Czech Republic; ^2^ Department of Ophthalmology Hospital Kralovske Vinohrady Prague Czech Republic; ^3^ Eye Center Prague Prague Czech Republic; ^4^ Faculty of Medicine Charles University Hradec Kralove Czech Republic cuni.cz; ^5^ Department of Ophthalmology Military University Hospital Prague Czech Republic uvn.cz; ^6^ First Faculty of Medicine Charles University Prague Czech Republic cuni.cz; ^7^ Eye Clinic Rosenheim Rosenheim Germany; ^8^ Medical Faculty University of Freiburg Freiburg Germany uni-freiburg.de

**Keywords:** aberration-neutral, C-loop IOL, hydrophobic, multifocal, presbyopia

## Abstract

**Purpose:**

To evaluate the effectiveness and safety of a new trifocal intraocular lens (IOL) in patients with age‐related cataracts 12 months after surgery.

**Methods:**

This prospective, noncomparative, multicenter study included 122 patients (244 eyes) undergoing cataract surgery with bilateral implantation of the AT ELANA 841P. At 12 months postoperatively, refraction and binocular visual acuity (VA) at distance (4 m), intermediate (80 cm), and near (40 cm) were measured. Visual symptoms and spectacle independence were subjectively evaluated. Safety assessment included the rate of ocular adverse events (AEs), posterior capsule opacification (PCO), IOL tilt and decentration, and glistening formation.

**Results:**

At 12 months postoperatively, most eyes (90.6%) were within ± 0.5 D spherical equivalent (SE) refraction, and almost all (99.6%) were within ± 1.0 D. Overall, 100% of patients achieved binocular uncorrected VA of 0.2 logMAR or better at distance. Performance at intermediate and near was 98.4% and 88.5%, respectively. All patients were spectacle‐free at far and intermediate distances (100%), and 88.5% were spectacle‐free for near tasks; over 80% reported no visual symptoms. The eye‐related AE rate was low, with mild‐to‐moderate intensity. Up to 12 months, 91.7% of the eyes had no PCO, while 3.2% were treated with posterior capsulotomy. IOL tilt was not observed; only a small number of decentration cases were monitored, and these were clinically insignificant (< 1 mm) at the final visit. At 12 months, the IOL showed no glistening (97.5%) or traces only.

**Conclusion:**

Implantation of the AT ELANA 841P provided reliable refractive and visual outcomes, high rates of spectacle independence, and corrected and uncorrected VA at all distances. The IOL exhibited a favorable safety profile over 12 months, characterized by minimal visual symptoms, low PCO, no glistening (or only trace), and a stable position in the eye.

**Trial Registration:** ClinicalTrials.gov identifier: NCT06247683

## 1. Introduction

Multifocal intraocular lens (IOL) implantation has been extensively demonstrated as safe and effective in restoring visual function in patients with cataracts and patients with presbyopia. Although trifocal IOLs can restore vision across a long range of distances, they are generally associated with visual symptoms when compared to monofocal IOLs [1–4].

A novel hydrophobic acrylic trifocal lens, AT ELANA 841P, developed on the well‐established CT LUCIA platform, was recently introduced in select markets. The C‐loop lens integrates a nonapodized refractive–diffractive optic surface.

The optical functions of the IOL have been investigated in several laboratory studies, providing reliable preclinical metrics that can predict the IOL postoperative performance. Preclinically, AT ELANA 841P demonstrated its trifocal properties with good optical performance across various distances, independent of spectral and spherical aberration conditions, resulting in good simulated visual acuity (VA) [5, 6]. When compared to its hydrophilic counterpart, AT LISA tri 839MP, AT ELANA 841P showed comparable optical quality regarding visual range and photic phenomena. Hydrophobic material did not seem to affect the severity of dysphotopsias [7]. Furthermore, using the VirtIOL device, an optical bench system allowing virtual IOL implantation on healthy individuals without cataract, Tecnis Synergy (J&J Vision), AcrySof IQ PanOptix (Alcon Laboratories), and AT ELANA 841P were evaluated for photic phenomena perception. The study showed that the starburst pattern size was significantly larger for Tecnis Synergy than the other two IOLs and that AT ELANA 841P was preferred in a night scene with a glare source [8].

Postsurgical IOL tilt and decentration may affect visual quality. IOL susceptibility to misalignment depends on the nature of aberration correction [9]. AT ELANA 841P is based on an aspheric aberration‐neutral optic designed to be less sensitive to IOL tilt and decentration [10]. In the laboratory, AT ELANA 841P was tested under varying spectral and corneal spherical aberration conditions and showed tolerance to decentration up to 1 mm [11].

More recently, clinical evidence collected from patients with cataracts bilaterally implanted with AT ELANA 841P showed that 84% were within ± 0.5 D of spherical equivalent (SE) refraction, and almost all patients were within ± 1.0 D at 4–6 months postsurgery [6, 12, 13]. The mean binocular defocus profile showed a continuous range of 0.1 logMAR or better vision from distance (+0.50 D) to near (−2.5 D) [6, 12, 13]. The mean monocular VA was 0.2 logMAR or better across the whole defocus range (from +1.50 D to −3.00 D) [13, 14]. Contrast sensitivity (CS) tested under various light conditions was within the normal range distribution of subjects aged 40–49 years [6, 12, 13]. Most patients (85%) did not report visual symptoms, whereas 15% experienced a halo of moderate intensity [6]. Patient satisfaction was high [13, 14].

Nonetheless, to fully assess IOL performance, longer follow‐up data on photic phenomena, axial stability, posterior capsule opacification (PCO) incidence, and IOL‐related adverse events (AEs) are needed. This study reports the visual and safety outcomes of patients with cataracts bilaterally implanted with AT ELANA 841P IOLs with a follow‐up of 12 months.

## 2. Materials and Methods

### 2.1. Study Design and Patients

This prospective, noncomparative, multicenter clinical investigation evaluated the safety and efficacy of the AT ELANA 841P IOL in patients with bilateral, age‐related cataracts over a 12‐month follow‐up period. Conducted across 11 sites in the Czech Republic, Germany, and Slovakia, the trial adhered to the ethical principles outlined in the Declaration of Helsinki, complied with Good Clinical Practice (ISO 14155), and followed all applicable local and national regulations governing clinical studies involving human subjects. Regulatory approvals were obtained from relevant Competent Authorities and Ethics Committees/Institutional Review Boards (NMB 31608/2022 in the Czech Republic; 2022‐620‐f‐S in Germany; and approval without a reference number in Slovakia). Prior to enrollment, patients were thoroughly informed about the potential risks, benefits, and constraints of participating in the study and gave written informed consent.

Eligible participants included adults of any gender with clinically significant bilateral age‐related cataracts and no major ocular pathologies. Participants were suitable for implantation of a posterior chamber trifocal IOL and had potential postoperative binocular CDVA of 0.2 logMAR or better, preoperative corneal astigmatism < 1.0 D and an IOL power requirement within 0.0 to +34.0 D. Exclusion criteria included systemic or ocular conditions that could increase surgical risk or affect study outcomes, including clinically significant corneal abnormalities, prior intraocular or corneal/refractive surgeries, degenerative visual disorders, use of systemic or ocular medications that could impact vision, and any ocular conditions likely to predispose the patient to future complications, as determined by the investigator’s medical judgment.

All patients received bilateral implantation of AT ELANA 841P following phacoemulsification. The interval between first and second eye surgery was 1–15 days.

### 2.2. IOL

AT ELANA 841P (Carl Zeiss Meditec AG) is a hydrophobic, acrylic IOL (refractive index of 1.49, measured at 35°C and at a wavelength of 589 nm) that has less than 1% water content, a UV light‐absorbing component, and a periodate‐oxidized heparin coating that has no pharmacological, immunological, or metabolic effects. The one‐piece, C‐loop, posterior chamber IOL, fully preloaded in the single‐use BLUESERT injector, is available from 0.0 to 34.0 D. The IOL overall diameter measures 13 mm, and the optic diameter is 6 mm. The optic is aberration‐neutral and trifocal over the full optic zone, with +1.66 D intermediate addition and +3.33 D near addition. The C‐loop lens integrates an entire refractive–diffractive optic surface and distributes light in a pupil‐independent manner to three foci (47.5% of light is allocated to far, 17.5% to intermediate, and 35% to near focus). The IOL posterior side features a continuous 360° sharp edge (including the optic–haptic–junction region) designed to inhibit early lens epithelial cell migration and lower the risk of PCO development.

The calculation of the IOL power for each patient’s eye was predominantly performed using the IOLMaster 700 (Carl Zeiss Meditec AG); one site used the IOLMaster 500. The recommended A‐constant was sourced from the IOL Con website (https://www.iolcon.org/). The “Barrett TK Universal II” formula was most frequently employed, though alternative formulas were allowed if anticipated to provide improved visual outcomes. In all instances, the target refraction was set to emmetropia (+0.00 D/plano).

### 2.3. Surgical Procedure

Standard small‐incision phacoemulsification cataract extraction was performed in all patients (complete procedure described in Janeková et al. [12]). There were no other specific medical or surgical procedures required. Intracameral antibiotics at the end of surgery, postoperative treatment, and medication were given according to the routine procedure in each study site.

### 2.4. Preoperative and Postoperative Assessments

All patients underwent a thorough preoperative assessment including a complete medical history, slit lamp and fundus examination, objective and subjective refraction, determination of the dominant eye, measurement of monocular and binocular uncorrected visual acuity (UDVA) and CDVA, biometry, pupil size, and intraocular pressure measurement.

Subjects were examined postoperatively at Day 1 and Week 1 following each eye and then for both eyes at 1 month, 3 months, 4–6 months, and at 12 months (330–420 days after the surgery of the second eye).

Clinical outcomes measured at 12 months included binocular UDVA and CDVA, uncorrected intermediate visual acuity (UIVA), distance‐corrected intermediate visual acuity (DCIVA), uncorrected near visual acuity (UNVA), distance‐corrected near visual acuity (DCNVA), subjective sphere (SPH), cylinder (CYL), and SE refraction. Optical and visual symptoms and spectacle independence were assessed with nondirected questioning. Safety was assessed by monitoring the IOL position (tilt and decentration), incidence of AEs, device deficiency, need for secondary surgical intervention, PCO with Nd:YAG treatment, and presence of glistening.

Binocular corrected and uncorrected VA was measured with an automated ETDRS chart using the Clinical Trial Suite (CTS) (M&S Technologies, Inc., Niles, IL, USA) that provided descending logMAR charts with 100% contrast and proportionally spaced SLOAN letters at the following distances: far 400 ± 12 cm; intermediate 80 ± 2 cm; near 40 ± 1 cm. The luminance of the charts was standardized at 85 cd/m^2^. SPH, CYL, and SE refraction were assessed at a defined distance of 4 m (±12 cm). Thus, the infinite SE refraction was obtained by adding − 0.25 D to the sphere component once the subjective refraction was measured at 4 m.

Nondirected questioning was conducted to inquire about the patients’ binocular visual symptoms, such as halo, glare, starburst, distortion, double images, and other phenomena. Visual symptoms were categorized and graded as mild, moderate, or severe. Nondirected questioning was also employed to evaluate patients’ use of spectacles and the distances for which spectacle correction was needed.

Slit‐lamp examination was used to evaluate IOL tilt and decentration. Decentration was defined as the distance in millimeters between the IOL optical axis and the center of the pupil, and its direction was recorded as nasal, temporal, superior, or inferior. IOL tilt was estimated using the iris plane as a reference and graded as none, slight (≤°5°), or significant (>°5°). The occurrence and incidence of AEs, device deficiency, and need for secondary surgical intervention were also recorded according to good clinical practice standards.

PCO was subjectively evaluated by the investigator during slit‐lamp examination using the following intensity scale: none (0), normal posterior capsule with no area of opacity; trace (+1), some loss of transparency involving the posterior capsule; mild (+2), mild loss of transparency with cloudiness extending through most of the posterior capsule (few Elschnig pearls may be present); moderate (+3), moderate loss of transparency with difficulty visualizing the retina (multiple Elschnig pearls may be present); and severe (+4), complete posterior capsule opacification with inability to see the retina (confluent Elschnig pearls and fibrous scarring may be present). Eyes with PCO were categorized by clinical significance: (a) clinically nonsignificant PCO, including fibrosis and proliferation of lens epithelial cells observed by slit‐lamp biomicroscopy, with no apparent subjective or objective VA change; (b) clinically significant PCO with VA changes (subjective or objective) not requiring a secondary capsulotomy; and (c) clinically significant PCO requiring Nd:YAG capsulotomy. The necessity of an Nd:YAG laser treatment was based on patient‐reported complaints and/or a VA loss of ≥°0.2 logMAR attributable to PCO. Following Nd:YAG treatment, binocular CS was measured at 1.5, 3, 6, 12, and 18 cpd spatial frequencies, under mesopic (3 cd/m^2^), mesopic with glare, and photopic with glare (85 cd/m^2^) conditions. The methodology for CS measurement and results of this cohort at 4–6 months were previously presented in Janeková et al. (2025) [12]; in the present manuscript, CS is reported for the post‐Nd:YAG subgroup only.

Finally, IOL glistening was evaluated during slit‐lamp examination. After pupil dilation, the number of glistenings was counted with the slit‐lamp beam set at 10.0 mm × 2.0 mm. Glistening density was then graded as follows: ≤°10 glistenings/field (trace or less), 11–20 glistenings/field (Grade +1), 21–30 glistenings/field (Grade +2), 31–40 glistenings/field (Grade +3), and >°40 glistenings/field (Grade +4).

### 2.5. Statistical Analysis and Sample Size

Sample size calculation was based on the assessment of the primary objective (mean monocular CDVA at 4–6 months is ≤ 0.02 logMAR) described in a first open‐access publication, Janeková et al. 2025 [12]. As the primary objective tested in a confirmatory manner was achieved and the null hypothesis could be rejected, all performance and safety secondary endpoints presented in this manuscript were tested in an exploratory manner.

Statistical analysis was performed using SAS Version 9.4 (SAS Institute Inc., Cary, USA), with descriptive statistics summarizing categorical variables as numbers and percentages, and continuous variables as means ± SD and ranges. Additionally, VA assessments were provided with two‐sided 95% confidence intervals and 95% exact confidence intervals (Clopper–Pearson) for the predefined AEs (ISO 11979‐7: 2018 Annex E). A *p*‐value < 0.05 was considered statistically significant.

## 3. Results

### 3.1. Patient Demographics and Preoperative Measurements

The study enrolled 135 subjects. Two withdrew consent before surgery and seven failed screening (including 1 identified after IOL implantation), resulting in 126 eligible patients (57.1% female) with a mean age of 63.3 ± 8.9 years (ranging from 33 to 81 years). The subjects were treated for mild‐to‐moderate cataract opacity (nuclear opacity NO1/NO2 in 73.4% of the eyes and cortical opacity C1/C2 in 76.6% of the eyes, according to the LOCS III grading scale [15]). Complete patient demographics were presented in Janeková et al. (2025) [12]. All patients were bilaterally implanted with the AT ELANA 841P IOL, with a mean spherical IOL power of 22.02 ± 2.85 D (ranging from 14.00 to 31.50 D). Preoperatively, subjective SE refraction was 0.21 ± 2.09 D, sphere was 0.62 ± 2.05 D, CYL was −0.32 ± 0.46 D, mean binocular UDVA was 0.29 ± 0.25 logMAR, and mean binocular CDVA was 0.05 ± 0.14 logMAR (Table 1). At the 12‐month postoperative visit, data from 122 patients (244 eyes) were available (4 patients did not want to continue the study after the 4‐ to 6‐month visit).

**TABLE 1:** Preoperative and postoperative refractive outcomes and binocular visual acuity at 12 months after bilateral implantation of AT ELANA 841P shown as means ± standard deviation (SD), two‐sided 95% confidence interval [95% CI], and ranges.

Refraction (D)	
Preoperatively	
Subjective SE^∗^	0.21 ± 2.09 (−7.50 to 5.88)
Subjective SPH	0.62 ± 2.05 (−7.25 to 6.50)
Subjective CYL	−0.32 ± 0.46 (−3.00 to 0.00)
Postoperatively (12 months)	
Subjective SE^∗^	−0.09 ± 0.34 (−1.13 to 0.75)
Subjective SPH	0.27 ± 0.35 (−0.75 to 1.25)
Subjective CYL	−0.22 ± 0.31 (−1.25 to 0.00)

**Visual acuity (logMAR)**	

Preoperatively	
UDVA	0.29 ± 0.25 [0.25; 0.33] (−0.12 to 1.48)
CDVA	0.05 ± 0.14 [0.02; 0.07] (−0.28 to 0.68)
Postoperatively (12 months)	
UDVA	−0.08 ± 0.08 [−0.09; −0.06] (−0.28 to 0.20)
CDVA	−0.11 ± 0.08 [−0.13; −0.10] (−0.30 to 0.24)
UIVA (80 cm)	0.02 ± 0.08 [0.00; 0.03] (−0.18 to 0.22)
DCIVA (80 cm)	0.02 ± 0.09 [0.00; 0.03] (−0.20 to 0.30)
UNVA (40 cm)	0.07 ± 0.10 [0.05; 0.09] (−0.20 to 0.36)
DCNVA (40 cm)	0.04 ± 0.11 [0.02; 0.06] (−0.20 to 0.28)

*Note:* SE∗, spherical equivalent (adjusted to optical infinity by adding −0.25 D to the sphere component once the subjective refraction was measured at 4 m).

Abbreviations: CDVA, corrected distance visual acuity; CYL, cylinder; DCIVA, distance‐corrected intermediate visual acuity; DCNVA, distance‐corrected near visual acuity; SPH, sphere; UDVA, uncorrected distance visual acuity; UIVA, uncorrected intermediate visual acuity; UNVA, uncorrected near visual acuity.

### 3.2. Objective Outcomes

#### 3.2.1. Refractive Outcomes

At 12 months postoperatively, the AT ELANA 841P IOL demonstrated reliable refraction. Most eyes (90.6%) were within ± 0.50 D SE refraction, and almost all (99.6%) were within ± 1.00 D. One implanted eye showed a slight myopic outcome with a SE refraction of −1.13 D, which was slightly below the cutoff value of −1.00 D. More than half of the eyes (53.7%) were within ± 0.25 D SE refraction (Figure 1). The mean subjective SE refraction (−0.09 ± 0.34 D) and CYL refraction outcomes (−0.22 ± 0.31 D) were within ±0.25 D. The mean refractive sphere was 0.27 ± 0.35 D (Table 1).

**FIGURE 1:**
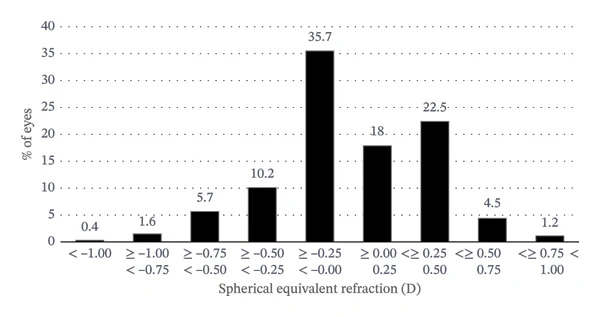
Distribution of spherical equivalent refraction at 12 months postoperatively.

#### 3.2.2. VA Outcomes

At 12 months postoperatively, patients achieved good binocular VA at all distances (data are summarized in Table 1 and Figure 2).

**FIGURE 2:**
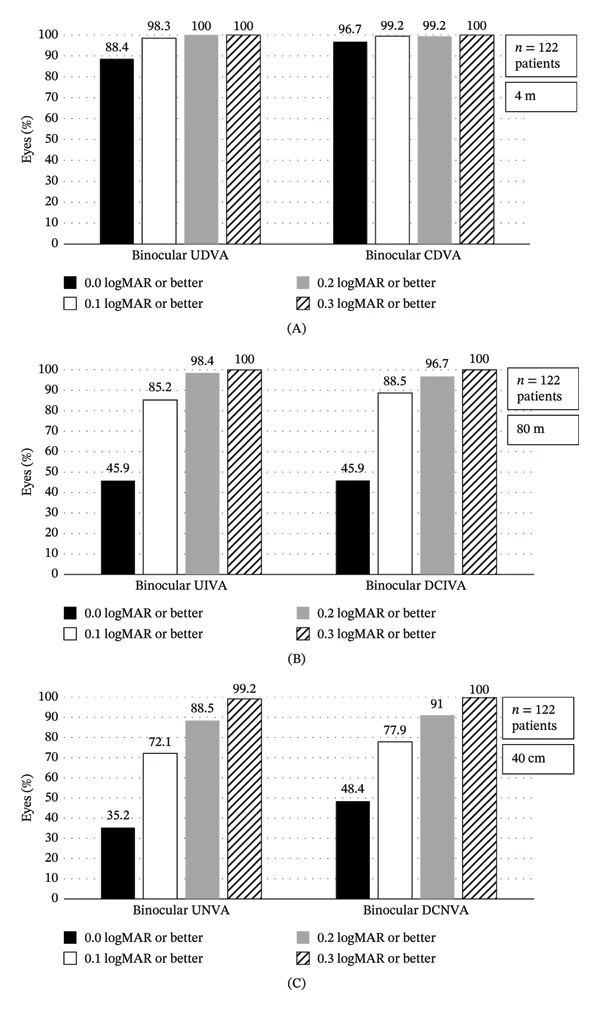
Distribution of binocular visual acuity in patients implanted with AT ELANA 841P at 12 months postsurgery with uncorrected distance visual acuity (UDVA) and corrected distance visual acuity (CDVA) (A), uncorrected intermediate visual acuity (UIVA) and distance‐corrected intermediate visual acuity (DCIVA) (B), uncorrected near visual acuity (UNVA), and distance‐corrected near visual acuity (DCNVA) (C).

At far distance, most patients (98.3%) reached binocular UDVA of 0.1 logMAR or better and 100% reached binocular UDVA of 0.2 logMAR or better. Almost all patients (> 99.2%) had binocular CDVA of 0.1 logMAR and 0.2 logMAR or better. Only one patient did not achieve CDVA of 0.2 logMAR due to severe dry eye (Figure 2, A). Mean binocular CDVA and UDVA were ‐0.11 ± 0.08 logMAR for a two‐sided 95% CI of −0.13 to −0.10 logMAR and ‐0.08 ± 0.08 logMAR for a two‐sided 95% CI of −0.09 to −0.06 logMAR, respectively. Mean UDVA gained almost 4 lines from baseline. At 12 months, CDVA was equal to or better than preoperative CDVA in 92.6% of patients (safety index) and 83.5% showed equal or better postoperative UDVA than preoperative CDVA (efficacy index).

At intermediate distance (80 cm), more than 96% of patients reached UIVA and DCIVA of 0.2 logMAR or better. Binocular UIVA categorization followed DCIVA categorization very closely (Figure 2, B). There was no difference between mean binocular UIVA and DCIVA (∼0.02 ± 0.08 logMAR [95% CI of 0.00–0.03], Table 1).

At near distance (40 cm), more than 88% of the patients achieved UNVA and DCNVA of 0.2 logMAR or better (88.5% and 91%, respectively) (Figure 2, C). Mean binocular UNVA was 0.07 ± 0.10 logMAR for a two‐sided 95% CI of 0.05–0.09 logMAR, and DCNVA was 0.04 ± 0.11 logMAR for a two‐sided 95% CI of 0.02–0.06 logMAR (Table 1).

In most cases, the cumulative binocular VA profiles observed at 12 months postoperatively were very similar to the ones observed at 4–6 months, strongly suggesting stable visual outcomes over time.

### 3.3. Subjective Outcomes

#### 3.3.1. Eye and Visual Symptoms Patient‐Reported Outcomes

Nondirected patient reports of vision quality showed that more than 80% were not disturbed by any type of visual symptoms at 12 months postoperatively. Overall visual symptoms frequency decreased over time and were reported by 37.8% of patients at 1 month, 34.6% at 4–6 months, and 18.9% at 12 months. The most frequently reported visual symptoms were halos, followed by glare. One patient encountered starburst (mild) from 1 month up to 12 months postoperatively. Distortion (mild) was reported by one patient only at 1 month. There were no double images and no severe types of visual symptoms. The distribution of halos and glare severity decreasing over time is shown in Figure 3. Halos (mostly mild) were experienced by 23.6% of patients at 1 month, 25.2% at 4–6 months and decreased to 10.7% at 12 months. Likewise, glare (mostly mild) declined from 7.1% at 1 month to 3.9% at 4–6 months and further to 3.3% at 12 months.

**FIGURE 3:**
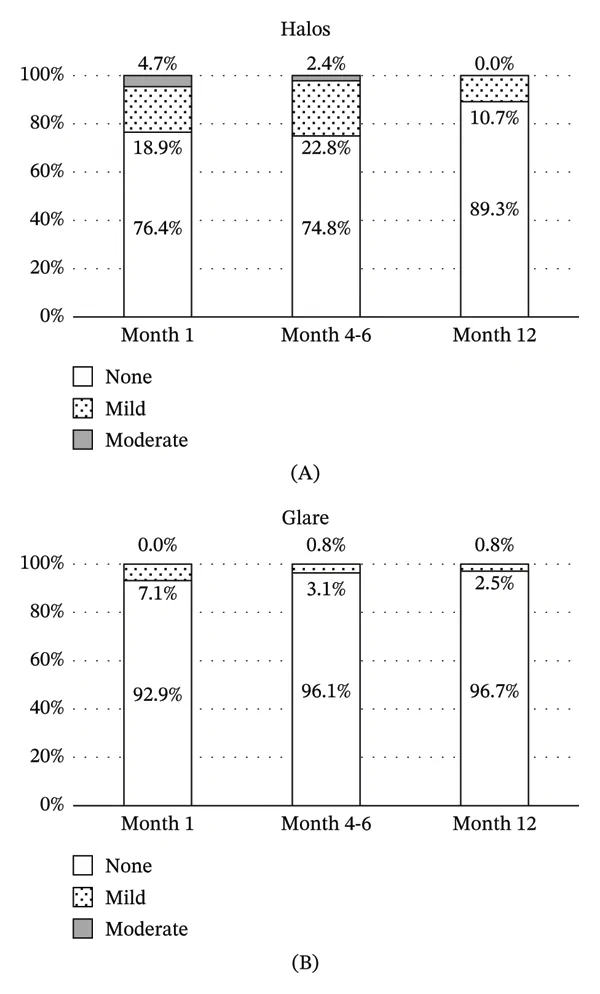
Distribution of visual symptom severity, halos (A) and glare (B), in patients implanted with AT ELANA 841P over 12‐month follow‐up.

Other symptoms such as ocular dryness (mild) were experienced by 12.6% at 1 month, 7.1% at 4–6 months, and 4.1% at 12 months. Mild‐to‐moderate ocular pain was seldom reported throughout follow‐up.

#### 3.3.2. Spectacle Independence

All patients (100%) were spectacle‐free for far and intermediate distances; 11.5% reported wearing glasses for near distance.

### 3.4. Safety Outcomes

#### 3.4.1. IOL Tilt and Decentration

Table 2 summarizes IOL tilt and decentration outcomes. No IOL tilt was recorded from 1 month onward. One case (0.4%) of slight IOL tilt (2.0°) was found 1 week postoperatively that spontaneously resolved by 1 month.

**TABLE 2:** IOL tilt and decentration over 12‐month follow‐up shown as numbers (n) and percentages of eyes affected (%), decentration measurement (mm) shown as mean ± standard deviation (SD) with ranges, and direction of decentration as numbers (n) and percentages of eyes affected (%).

	IOL tilt		IOL decentration		
	*n* eyes (%)	*n* eyes (%)	Decentration (mm)	Direction *n* eyes (%)	
			mean ± SD (min; max)	Horizontal temporal	Vertical superior
Month 1	0 (0%)	5 (2.0%)^∗^	1.75 ± 0.50 (1.0 to 2.0)^∗^	4 (80%)^∗^	4 (80%)^∗^
Month 3	0 (0%)	3 (1.2%)	1.17 ± 0.29 (1.0 to 1.50)	3 (100%)	3 (100%)
Months 4–6	0 (0%)	4 (1.6%)	0.50 ± 0.0 (0.50 to 0.50)	4 (100%)	4 (100%)
Month 12	0 (0%)	4 (1.6%)	0.63 ± 0.25 (0.50 to 1.00)	4 (100%)	4 (100%)

^∗^At 1 month, 5 cases of IOL decentration were observed in 4 subjects, and 1 subject had decentration in both eyes, while the value and direction of decentration were missing for 1 subject presenting with an IOL subluxation in one eye.

Regarding IOL decentration, 5 cases of 1.0–2.0 mm of decentration were reported at 1 month (value missing for one subject presenting with an IOL subluxation in one eye) and 3 cases at 3 months. Upon further follow‐up, IOL decentration stabilized: 4 eyes showed decentration between 0.50 and 1.00 mm (mean value of 0.63 ± 0.25 mm at 12 months), while exhibiting good visual acuities 12 months postoperatively, suggesting that slight IOL decentration (< 1 mm) was not associated with an apparent reduction in VA. Direction of decentration was equally distributed with no relation to visual outcome.

#### 3.4.2. AEs

Table 3 reports the details of eye‐related AEs and device‐related AEs recorded for all subjects who received AT ELANA 841P IOL (*n* = 127). In total, 49 eye‐related AEs affected 25 patients (19.7%) during the 12‐month study. Almost all eye‐related AEs were of mild‐to‐moderate intensity. There was one case (0.8% [95% CI 0.0%–4.3%]) of secondary surgical intervention for IOL repositioning due to early postoperative IOL subluxation (resulting in reduced VA after surgery), for which the occurrence was within the acceptable safety and performance endpoint (SPE) rates recommended by ISO 11979‐7. Normal position of the IOL was confirmed at the 3‐month follow‐up visit. There were 3 cases (2.4% [95% CI 0.5 to 6.7]) of cumulative, nonpersistent, cystoid macular edema, which were within the recommended limits of the ISO SPE rate. Twelve eye disorder AEs (4.7%) were qualified as device‐related: halo vision (6), glare (4), lens decentration (1), and reduced VA postoperatively due to IOL subluxation (1). One case of an IOL haptic captured in the cartridge of the injector led to an IOL exchange during surgery, resolving without sequelae. No serious eye‐related AEs were encountered.

**TABLE 3:** Eye‐related AEs and device‐related AEs regarding all subjects implanted with AT ELANA 841P (*n *= 127) and followed up over 12 months, shown as number (n) and percentage of implanted subjects (%) affected by AEs and number of events (m).

	Overall eye‐related AEs		Device‐related AEs	
	*n* subjects (%)	m	*n* subjects (%)	m
Eye disorders	25 (19.7%)	49	6 (4.7%)	12
Dry eye	5 (3.9%)	9		
Cystoid macular edema^∗∗^	3 (2.4%)	5		
Ocular hypertension	3 (2.4%)	3		
Uveitis	2 (1.6%)	4		
Conjunctivitis	2 (1.6%)	2		
Lens dislocation	3^‡^ (2.4%)	3	1^∗^ (0.8%)	1
Halo vision	3 (2.4%)	6	3 (2.4%)	6
Conjunctivitis allergic	1 (0.8%)	2		
Eye irritation	1 (0.8%)	2		
Iridocyclitis	1 (0.8%)	1		
Iritis	1 (0.8%)	1		
Visual acuity reduced after surgery^∗∗^	1 (0.8%)	1	1 (0.8%)	1
Vitreoretinal traction syndrome	1 (0.8%)	1		
Glare	2 (1.6%)	4	2 (1.6%)	4
Corneal edema	2 (1.6%)	3		
Dacryostenosis acquired	1 (0.8%)	1		
Keratitis	1 (0.8%)	2		
Secondary surgical intervention^∗∗^	1 (0.8%)	1		
Repositioning due to subluxation	1 (0.8%)	1		
IOL exchange during surgery due to injection problem	1 (0.8%)	1	1 (0.8%)	1

^∗∗^Number of events below the recommended safety and performance endpoint (SPE) rates according to ISO 11979‐7.

^‡^2 decentrations; 1 subluxation.

^∗^1 decentration.

#### 3.4.3. PCO and Nd:YAG Rates

Up to 12 months postoperatively, 8.7% of all eyes implanted with the device (22/254 eyes) showed PCO distributed as traces (4.7%), mild (3.2%), and moderate (0.8%). No severe cases of PCO were reported. Furthermore, among eyes presenting PCO, 3.2% (8/254 eyes) were treated with Nd:YAG laser posterior capsulotomy after patient complaints and report of clinically significant PCO requiring Nd:YAG. No significant CDVA loss (≥ 0.2 logMAR) attributable to PCO was observed (Table 4).

**TABLE 4:** PCO and Nd:YAG rates up to 12 months postsurgery in all subjects implanted with AT ELANA 841P IOL (*n *= 127 subjects).

	Up to 12 months *n* eyes (%)
PCO	**22/254 (8.7%)**
Intensity	
Trace (+1)	12 (4.7%)
Mild (+2)	8 (3.2%)
Moderate (+3)	2 (0.8%)
Severe (+4)	0 (0%)
Clinical significance	
Clinically nonsignificant	14 (5.5%)
Clinically significant	0 (0%)
Clinically significant requiring YAG	8 (3.2%)
Significant CDVA loss^∗^	
No	22 (8.7%)
Yes	0 (0.0%)
Nd:YAG performed	**8/254 (3.2%)**
Patient‐reported complaint	8 (3.2%)

*Note:* Trace (+1): some loss of transparency involving the posterior capsule; Mild (+2): mild loss of transparency with cloudiness extending through most of the posterior capsule; Moderate (+3): moderate loss of transparency with difficulty visualizing the retina; Severe (+4): posterior capsule very opaque with inability to view the retina.

^∗^VA loss of ≥ 0.2 logMAR attributable to PCO.

#### 3.4.4. Glistening

At 12 months, 97.5% of eyes (238/244) had no glistening, while 2.5% (6/244) had traces (< 10 glistening/field under mydriasis observation).

## 4. Discussion

This study confirms that implantation of the AT ELANA 841P in patients with bilateral cataracts is safe and effective in restoring high‐quality vision and in providing high rates of spectacle independence.

At 12 months postoperatively, 90.6% of the eyes were within ± 0.50 D of infinity‐adjusted SE refraction and all but one eye (99.6%) were within ±1.00 D, thus confirming reliable refraction predictability. The “Barrett TK Universal II” formula was appropriate for AT ELANA 841P IOL power calculation. Additionally, the Z CALC Online IOL Calculator could be used.

Refractive outcomes were aligned with an efficacy index ≥ 1 in 83.5% of patients, indicating that a high number of surgeries successfully achieved the desired visual improvement without compromising safety as shown by a binocular safety index ≥ 1 in more than 92% of patients.

Regarding distance VA, 98.3% of patients reached binocular UDVA of 0.1 logMAR or better and all patients (100%) reached 0.2 logMAR or better. These results are comparable to the performance of established trifocal IOLs on the market [16–19]. However, published studies provide contextual benchmarks only, which should be interpreted cautiously due to cross‐study comparison limitations (differences in study design, testing distances, patient populations, and follow‐ups). At 6 months, 94.9% and 100% of patients bilaterally implanted with the AcrySof IQ PanOptix (*n* = 138) reached binocular UDVA of 0.1 and 0.2 logMAR or better, respectively, while 89.1% and 98.6% reached the same thresholds, respectively, when implanted with Tecnis Synergy (*n* = 138) [16]. In a large pooled analysis (*n* = 542 patients, 4–6 months), 92.1% reached 0.14 logMAR with the AcrySof IQ PanOptix [17]. Results were similar for bilateral implantation of the newest Clareon PanOptix IOL at 3 months [18] and at 12 months [19].

Binocular intermediate visual outcomes with AT ELANA 841P at 80 cm were favorable: 85.2% achieved UIVA of 0.1 logMAR or better, and 98.4% achieved 0.2 logMAR or better. Published data for other trifocal IOLs have reported similar intermediate outcomes [16, 17, 19, 20]. However, methodological differences preclude direct comparative conclusions; in particular, Kohnen et al. [17] and Bayhan et al. [20] used different VA thresholds and some of the other studies assessed intermediate vision at 60–66 cm rather than 80 cm.

At near (40 cm), 88.5% of patients implanted with AT ELANA 841P reached binocular UNVA of 0.2 logMAR or better. Published findings for other trifocal IOLs also suggest good near performance [16, 19].

Moreover, at 12 months postoperatively, 100% of patients implanted with AT ELANA 841P were spectacle‐free for far and intermediate vision, 88.5% did not need glasses for any type of daily activity, and 11.5% used them for activities that required near vision. At 3 months, a high spectacle independence rate (85.1%) was also reported for AT ELANA 841P in another study [21]. Published reports of other trifocal IOLs have described similarly high levels of spectacle independence [22–24], although differences in patient counseling, questionnaire format, and definitions of spectacle use limit direct comparison.

Although trifocal lenses provide excellent uncorrected VA at all distances, with spectacle independence close to 100%, a generally known drawback is the decrease in CS and the reportedly high rates of visual symptoms.

Halo, usually of mild‐to‐moderate intensity, is among the most frequently reported visual symptoms after implantation of trifocal IOLs [20, 23, 25, 26]. In the present study, more than 80% of patients reported not being disturbed by visual symptoms at 12 months. Among reported symptoms, halo was more common than glare, starburst, or distortion, and both frequency and severity decreased over time. At 12 months, halos were reported by 10.7% and glare by 3.3% of patients, most often of mild intensity. Nevertheless, these findings were collected through nondirected questioning rather than a validated patient‐reported outcome questionnaire and thus should be interpreted cautiously, as the approach may have led to underreporting of minor but clinically relevant visual symptoms.

Complementarily, additional published evidence for AT ELANA 841P confirmed minimal reports of visual symptoms as early as 3 months postoperatively. In a prospective case series of 40 patients, 85% did not report visual symptoms when shown representative images of halos and glares, while 10% experienced “moderate” halos “most of the time” and 5% had “slightly bothersome” halos “very rarely” [6]. Using extensive patient‐reported outcome questionnaires (AIOLIS questionnaire), Auffarth et al. (2026) [21] reported that 59.6%–100% of patients (*n* = 47) reported “never or sometimes” experiencing visual symptoms such as snowballs, halos, starburst, glare, distortion, hazy vision, and floaters. At 6 months, patients (*n* = 10) did not complain of bothersome phenomena when using the web‐based photic phenomena assessment (VisionSimulations.com platform) [27].

Furthermore, in this study, at 12 months, 97.5% of the eyes had no glistening and 2.5% had traces, based on the Christiansen scale of scoring [28]. These results are comparable to the “glistening‐free” Clareon PanOptix lens made of the new hydroxyethyl methacrylate (HEMA) material [19, 23, 29, 30].

The development of PCO is another factor that impacts visual outcomes over time. By 12 months postoperatively, 8.7% of the eyes showed PCO of mostly trace to mild intensity (2 eyes developed moderate PCO). These findings seem to correspond with findings of similar hydrophobic, trifocal IOLs. For the same time period, Rementeria‐Capelo et al. [23] reported 15% and 6% moderate PCO for eyes implanted with AcrySof IQ PanOptix and Clareon PanOptix, respectively. At 6 months, Sahin et al. [31] reported 11.6% PCO with AcrySof IQ PanOptix, although not clinically significant, and Dick et al. [25] reported 7.7% mild‐to‐moderate PCO at 3 months for the same device. Moshirfar et al. [32] reported 10.1% Nd:YAG treatment in patients with significant PCO within the first year following AcrySof IQ PanOptix implantation.

Moreover, Zhang et al. (2025) [33] recently showed that mild PCO initially affects near VA in patients with trifocal IOLs and may lead to decreased vision and CS. Patients with trifocal IOLs are thus more sensitive to PCO than patients with monofocal IOLs.

In our study, at 12 months postoperatively, 3.2% of patients had clinically significant PCO requiring YAG, without any significant loss of CDVA (loss of ≥ 0.2 logMAR attributable to PCO), but with patient‐reported complaints. Binocular far CS was reassessed at 12 months in four subjects who underwent Nd:YAG laser capsulotomy for PCO (in one or both eyes) after the 4‐ to 6‐month visit. Results showed that following Nd:YAG treatment, CS at all spatial frequencies and in all light conditions tested (photopic with glare, mesopic with and without glare) was within the reference range reported for phakic subjects aged 40–49 years exposed to daylight with and without glare [34] (Supporting Figure 1). This subgroup finding should be interpreted as descriptive only because of the small sample size. Importantly, binocular CS of the complete patient cohort was tested at the 4‐ to 6‐month visit and reported in a first article (Janeková et al. 2025 [12]). CS was found to fall within the normal range for all light conditions tested.

Implantation of the IOL did not reveal unexpected safety concerns within the observation period. The number of eye‐related AEs was low, with AEs of mostly mild‐to‐moderate intensity. All AEs recorded during the study were below the SPE rates recommended by the ISO standard. No endophthalmitis, hypopyon, lens dislocation from the posterior chamber, pupillary block, retinal detachment, or persistent events were reported. Moreover, the IOL was stable in the eye. IOL decentration of up to 1 mm was reported for 4 eyes at 12 months and was not associated with an apparent reduction in VA in these cases.

Patient satisfaction was not assessed in the present study. However, additional published data for AT ELANA 841P reported Catquest‐9SF outcomes at 3 months with 100% of patients (*n* = 23) being “fairly” or “very satisfied” with their vision and 100% having “no difficulty or some difficulty” in their everyday life due to their sight [13]; at 6 months, 90% of patients reported no vision‐related difficulties in daily life, and all were “very satisfied” postoperatively [27]. Moreover, Mojzis et al. [27] qualified the visual quality as favorable and consistent with improvement of aberrometry metrics at 6 months.

Finally, this study has several limitations. First, it was a noncomparative investigation without a randomized or matched comparator arm. Second, the cohort included patients with a relatively young mean age. Consequently, the findings may not be fully representative of the broader cataract population seen in routine clinical practice, thereby limiting the generalizability of the results. Third, patient‐reported outcomes were collected by nondirected questioning rather than with validated questionnaires. This resulted in limited spectacle‐use data; specifically, the study did not capture the type of near‐vision activities requiring spectacles or the frequency at which spectacles were used. Likewise, nondirected questioning may have led to underreporting minor but clinically relevant visual symptoms. Validated questionnaires such as the NEI VFQ‐25 questionnaire, including questions targeting visual phenomena, could provide a more comprehensive understanding of patient satisfaction and visual quality following cataract surgery. Further clinical trials with the lens are expected, notably to assess full visual range performance together with a detailed examination of patient satisfaction.

## 5. Conclusion

In conclusion, implantation of the AT ELANA 841P in patients with bilateral cataract was associated with reliable refractive and visual outcomes and high rates of spectacle independence. The IOL demonstrated a favorable safety profile, with low rates of mostly mild visual symptoms, low incidence of PCO, no glistening or traces, and stable positioning in the eye over 1 year.

## Funding

The study was supported by Carl Zeiss Meditec AG, Germany. In accordance with the regulatory objectives of the clinical study, the sponsor supported the study design, data management/statistical analysis, and manuscript development.

## Disclosure

The authors reviewed and approved all manuscript drafts and made the final decision to submit the manuscript for publication.

## Conflicts of Interest

The authors declare no conflicts of interest.

## Supporting Information

Additional supporting information can be found online in the Supporting Information section.

## Supporting information


**Supporting Information** Supporting Figure 1 Mean binocular contrast sensitivity of four patients following Nd:YAG treatment measured at 12 months. Light conditions included photopic with glare, mesopic without glare, and mesopic with glare. Background areas represent the normal range distribution of subjects aged 40–49 years in daylight with glare (light blue) and daylight without glare (gray hatched) conditions [34].

## Data Availability

The data that support the findings of this study are available from the corresponding author upon reasonable request. The data are not publicly available due to privacy or ethical restrictions.
