# Supplementary material for: A One‐Year Visual and Safety Evaluation of a Newly Available Trifocal Intraocular Lens in Patients With Bilateral Cataracts: Clinical Outcomes From a Prospective Study
Source: J Ophthalmol. 2026 Oct 5;2026:5786325. doi: 10.1155/joph/5786325 (PMC13639549; doi:10.1155/joph/5786325)
Supplement: Supplementary file 1 [file JOPH-2026-5786325-s001.pdf]

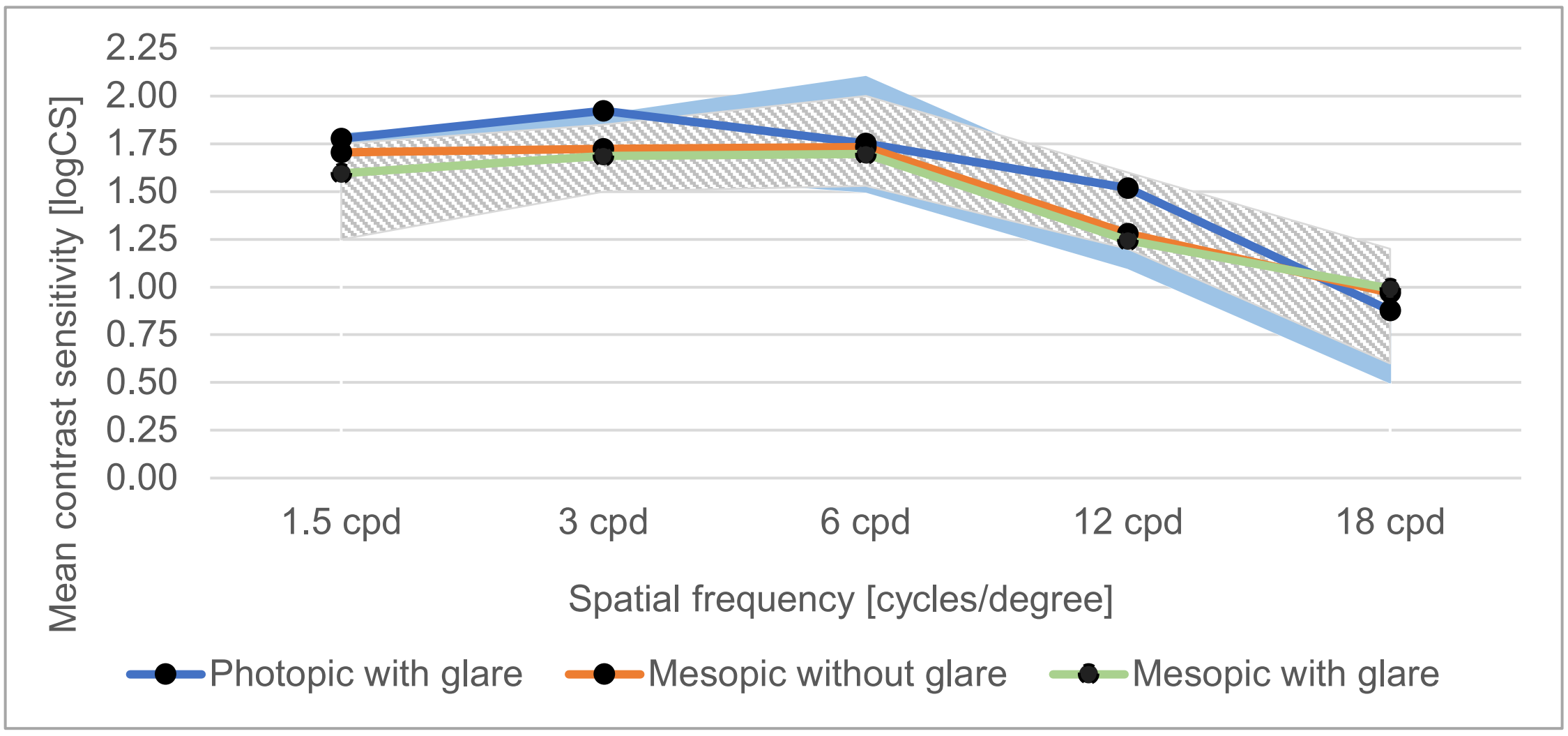


**Supplementary Figure 1** Mean binocular contrast sensitivity of four patients following Nd:YAG treatment measured at 12 months. Light conditions included, photopic with glare, mesopic without glare and mesopic with glare. Background areas represent normal range distribution of subjects aged 40-49 years in daylight with glare (light blue) and daylight without glare (gray hatched) conditions [34]
